# A Case Report of Epidural Hematoma After Traumatic Brain Injury

**DOI:** 10.21980/J8R059

**Published:** 2020-07-15

**Authors:** Ronald Goubert, Alisa Wray, Danielle Matonis

**Affiliations:** * University of California, Irvine, Department of Emergency Medicine, Orange, CA

## Abstract

An epidural hematoma (EDH) is a potentially life-threatening intracranial hemorrhage (ICH) that may require emergency neurosurgical intervention to prevent rapid clinical decline. This case report discusses the presentation, diagnosis and neurosurgical intervention of an adult male who presented unresponsive and was found to have a right-sided epidural hematoma along with a left-sided subdural hematoma requiring surgical placement of an extra-ventricular drain. Although most epidural hematomas are easily visible on non-contrast computed tomography (CT), it is important to be able to recognize key diagnostic findings that separate epidural hematomas from other types of intracranial hemorrhages. While many are managed without surgery, it is important to know the presentation, progression, and diagnostic criteria that may warrant neurosurgical intervention to combat the effects of an expanding hematoma and increasing intracranial pressure.

**Topics:**

Epidural Hematoma (EDH), Intracranial Hemorrhage (ICH), Traumatic Brain Injury (TBI).

**Figure f1-jetem-5-3-v22:**
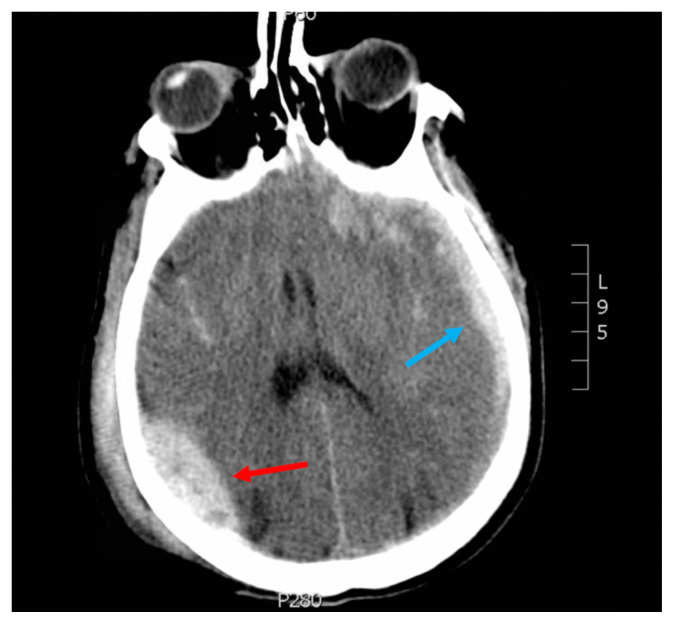


## Introduction

[Fig f1-jetem-5-3-v22]Head injury is the leading cause of death and disability in children and young adults.^1^ Of the different types of ICH, epidural hematomas (EDH) account for 2%.7–4% of all traumatic brain injuries; these are more likely to occur in the second decade of life.^2^ Although ICHs have a mortality of 40% within one month of presentation, EDHs are typically associated with better outcomes and a mortality of less than 10% if they are quickly identified and treated.^3^,^4^,^5^,^6^ These patients are also at an increased risk of developing long-term neuropsychological sequelae such as headaches, seizures, cognitive impairment, and depression.^7^ Early recognition and proper management of an EDH is critical in reducing morbidity and mortality associated with traumatic head injuries.

## Presenting concerns and clinical findings

A 57-year-old male with history of hypertension and seizures secondary to alcohol dependence presented as a trauma activation to the emergency department (ED) after being found down on the cement while working as a car mechanic. On initial presentation to the ED, the patient was awake but altered. He had a right parietal scalp hematoma with an overlying abrasion but no palpable skull fracture. His eyes were open, but he did not answer questions or follow commands giving a GCS of 10 (4-1-5). Both pupils were symmetric and reactive, and he appeared to be moving all extremities symmetrically. No other traumatic injuries were found. He required treatment for seizures both before and during the initial computed tomography (CT) scan.

## Patient Course

After the primary trauma survey, the patient was immediately taken to CT scan where he continued to have seizures. He was treated with intravenous lorazepam (4 mg) with cessation of seizure activity. The initial CT scan showed multiple ICHs, including a right EDH and a left-sided subdural hematoma. Neurosurgery was consulted for emergent surgical management. The patient was taken to the operating room for right craniotomy, evacuation of EDH, and placement of a right external ventricular drain (EVD). Neurosurgery also performed a left craniotomy for evacuation of the left subdural hematoma and placement of left frontal and left cerebral microdialysis catheters. While our patient was unfortunately lost to follow up, this case demonstrates a presentation of EDH that required surgical intervention based on both imaging and clinical presentation.

## Significant findings

Non-contrast CT head demonstrated a right sided EDH (red arrow) with overlying scalp hematoma, left-sided subdural hematoma (blue arrow), and bilateral subarachnoid hemorrhages. No skull fractures were noted.

## Discussion

An EDH occurs when there is an injury to a middle meningeal artery or vein, diploic vein, or venous sinus. An injury to one of these vessels will result in blood accumulation between the inner skull and the dura mater.^2^,^8^ Arterial injury, which occurs in 85% of EDHs, leads to a high pressure bleed that rapidly expands and compresses the surrounding brain parenchyma.^2^ Classically, an EDH is associated with a lucid interval after injury in which patients are asymptomatic for minutes to hours.^2^,^9^ During this time, blood collects in the epidural space before exerting mass effect on the surrounding brain tissue. Symptoms of an EDH typically present after an initial loss of consciousness and can include headache, dizziness, and/or neurological dysfunction.^6^ As more blood collects, the excess pressure causes pupillary abnormalities, hemiparesis, seizures, obtundation, and eventual herniation.^2^,^8^,^10^

An EDH is typically diagnosed utilizing CT imaging, which demonstrates a characteristic biconvex hyperdensity against the inner skull.^8^,^11^ Another diagnostic characteristic of an EDH is that the hemorrhage does not cross cranial suture lines as a result of the periosteal layer of the dura directly attaching to the sutures.^12^ It can, however, cross the midline of the skull.^12^ In addition, an overlying skull fracture is found in up to 95% of patients with an EDH.^13^,^14^ These differentiating characteristics are important considerations in early imaging when the full extent of injury is not yet apparent.

Early diagnosis and monitoring is crucial because several newer studies have demonstrated that hemorrhagic lesions may increase in size after initial presentation.^11^ This increase in size, known as progressive hemorrhagic injury (PHI), occurs in up to 50% of patients and can have severe consequences if it goes undiagnosed.^11^ Resulting PHI causes further compressive effects on surrounding brain parenchyma resulting in decreased cerebral perfusion pressure.^7^ Although many epidural hematomas can be managed conservatively, they can quickly progress and require emergent neurosurgical intervention.^2^,^14^ Indications for conservative management and serial CT imaging include an EDH that is less than 30cm^3^ with less than 15mm thickness, less than 5mm of midline shift, a GCS greater than 8, and no focal neurological deficits.^2^,^5^ The mainstay of conservative management is monitoring for clinical decline through frequent neurological exams and pupillometry, as well as control of blood pressure and blood glucose levels which could worsen cerebral ischemia.^15^ Other important considerations are sedation with artificial ventilation, elevating the head of the bed to 30°, and seizure prophylaxis.^15^ Even after conservative management, these patients require early follow-up imaging to monitor for PHI.^11^,^16^,^17^ All patients with an EDH necessitate emergent neurosurgical consultation since the decision for surgical versus conservative management is a complex decision based on both imaging and clinical criteria.^15^ In this case, emergent neurosurgical evacuation was necessary due to the patient’s imaging demonstrating a right-sided epidural hematoma that was greater than 15mm in thickness as well as the progressive worsening of his seizures.

In conclusion, epidural hematomas are traumatic intracerebral hemorrhages that are usually associated with an overlying skull fracture. Brisk bleeding from the middle meningeal artery or other large vessels can lead to rapid clinical deterioration. Although they are easily identified on most non-contrast CT images, key clinical characteristics help to differentiate them from other types of intracranial hemorrhages. Clinicians must be aware of these differences as well as the clinical and radiological signs that warrant timely neurosurgical intervention.

## Supplementary Information




