# Supplementary figures and images for: A Case Report of Epidural Hematoma After Traumatic Brain Injury
Source: J Educ Teach Emerg Med. 2020 Jul 15;5(3):V22–4. doi: 10.21980/J8R059 (PMC10332544; doi:10.21980/J8R059)

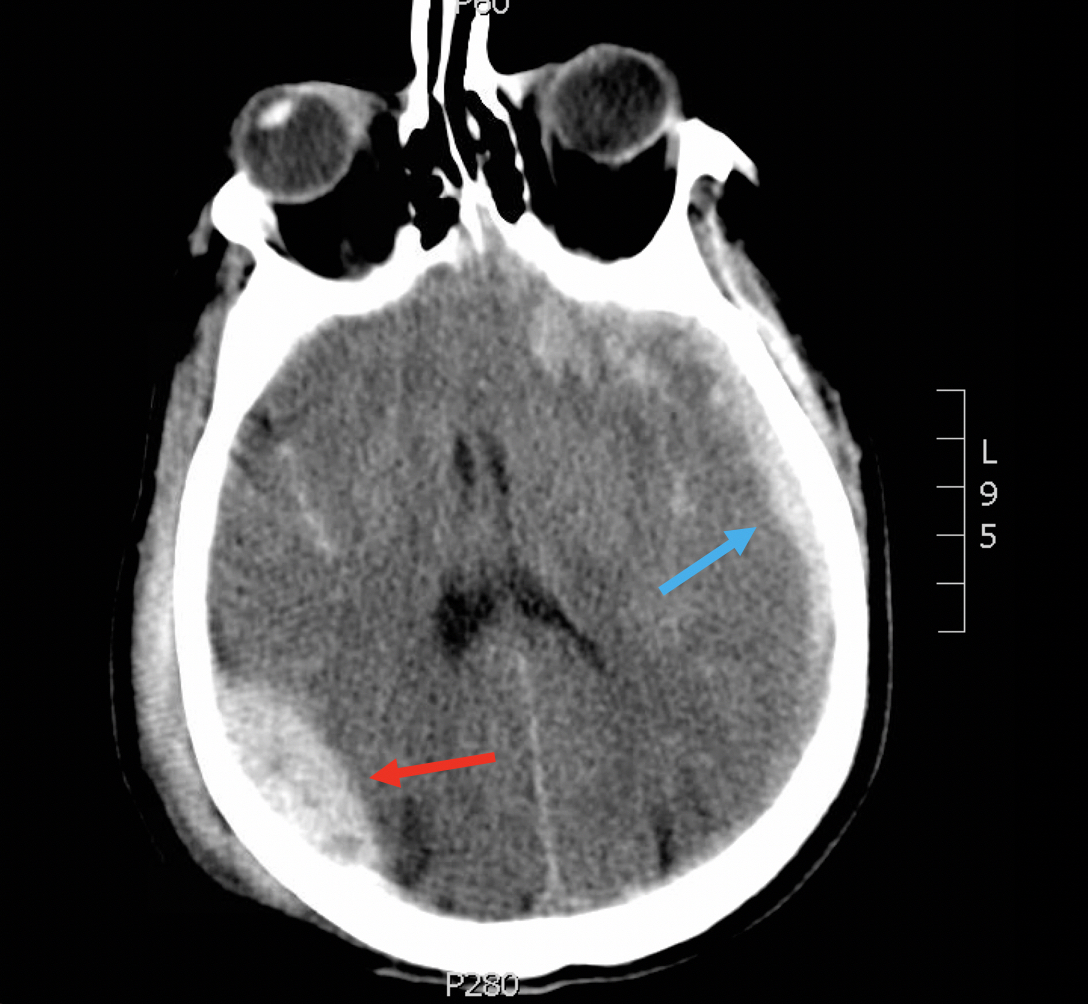

Supplement: Supplementary file 1 [file jetem-5-3-v22-supp1.jpg]

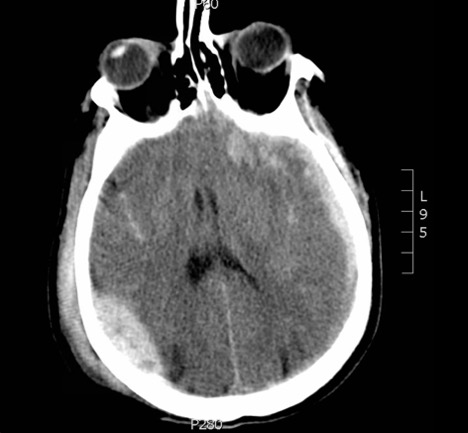

Supplement: Supplementary file 2 [file jetem-5-3-v22-supp2.jpg]
